# Forkhead Transcription Factor FOXP3 Upregulates CD25 Expression through Cooperation with RelA/NF-κB

**DOI:** 10.1371/journal.pone.0048303

**Published:** 2012-10-29

**Authors:** Cristina Camperio, Silvana Caristi, Giorgia Fanelli, Marzia Soligo, Paola Del Porto, Enza Piccolella

**Affiliations:** Department of Biology and Biotechnology “Charles Darwin”, University Sapienza of Rome, Rome, Italy; New York University, United States of America

## Abstract

Considerable evidence supports the prediction that CD25 is directly regulated by the forkhead transcription factor FOXP3. However, given that CD25 is normally upregulated in activated T cells, regardless of whether they express FOXP3, this issue has still to be definitively demonstrated. Here we describe that FOXP3, induced by CD28 signals in human CD4^+^CD25**^−^** T lymphocytes, synergizes with RelA on a regulatory region of *Cd25* promoter to mediate the transcriptional activation of *Cd25* gene. We found that a striking feature of this regulatory region is the presence of a κB site and of two tandem copies of a non-consensus FOXP3 binding site separated at 5′ ends by 19 nucleotides that allow FOXP3 and RelA binding to DNA and their physical interaction. The occupancy of the two FOXP3 binding sites in conjunction with RelA binding site occupancy allows FOXP3 to function as a positive activator of *Cd25* gene. Indeed mutations of both FOXP3 binding sites such as mutation of κB site on *Cd25* promoter abolished FOXP3 activatory functions. Moreover, FOXP3 mutation ΔE251, that compromises FOXP3 homotypic interactions, failed to trans activate *Cd25* promoter, suggesting that both FOXP3 DNA binding and dimerization are required to trans activate *Cd25* promoter. These findings identify a novel mechanism by which RelA and FOXP3 cooperate to mediate transcriptional regulation of target genes and characterize a region on *Cd25* promoter where FOXP3 dimer could bridge intramolecularly two DNA sites and trans activate *Cd25* gene.

## Introduction

The *FOXP3* gene that encodes the *forkhead* family transcription factor FOXP3 (forkhead box P3) has been identified as the master gene of regulatory T cells (T_reg_), a class of T cells that develops in the thymus and is essential for maintaining self-tolerance [1], [2], [3]. Mutations within the *FOXP3* gene in male infants result in a severe autoimmune syndrome termed IPEX (immune dysregulation, polvendocrinopathy, enteropathy, X-linked), characterized by defects in T_reg_ development and the consequent activation of autoreactive T cells [4], [5]. FOXP3 has distinct functional domains: a N-terminal domain required for transcriptional activation and repression, a central single C2H2 zinc finger with a not yet defined function (Znf), a leucin zipper-like motif (LZ) implicated in multimer formation and the carboxyl-termination forkhead domain (FKH) that mediates DNA-binding [6]. More recently, it has been proposed by modelling studies that FOXP3 molecules form a domain-swapped dimer through their FKH domains and the two dimers associate with each other through their Znf-LZ regions [7]. Analysis of FOXP3 function has shown that FOXP3 can not only repress but also induce gene expression. Indeed FOXP3 can negatively regulate transcription of some gene, such as *Il2*, but positively regulate others, such as *Cd25* and *Ctla4* [8], [9]. Several biochemical data indicate that FOXP3 does not function alone, but rather coordinates itself with various transcription factors, including NFAT, NF-κB, AP-1 and AML1/Runx1, to mediate transcriptional regulation of target genes [8], [9], [10], [11]. FOXP3 can also modulate gene expression through epigenetic mechanisms, such as histone acetylation and deacetylation [8], [12]. Moreover, FOXP3 functions can be further modified by post-translational modifications of FOXP3 induced by extrinsic cellular signals [12], [13], [14]. Given the complexity of the mechanisms involved in FOXP3 activity on target genes, regulation of FOXP3 function is still a relevant area of investigation.

Together with FOXP3, another highly specific marker for T_reg_ is CD25 [15], the essential component of the high-affinity IL-2R that is expressed on all activated T cells. The human *Cd25* promoter contains two important positive regulatory regions, PRRI (–276 to –244) and PRRII (–137 to –64), both required for mitogenic stimulation of CD25 [16]. NF-κB binding sites have been identified in the PRRI region and the classical NF-κB p50-p65 complexes have been described as the principal activators of CD25 transcription [17]. However, there is a wide evidence supporting FOXP3 as another activator of CD25. Ectopic expression of FOXP3 in CD4^+^CD25**^−^** naïve T cells can convert these cells to CD4^+^CD25^+^ T cells [18], [19]. FOXP3 can be recruited on specific binding site on *Cd25* promoter after T cell activation and mediate histone acetylation of the involved region [8], [9]. However, whether the binding of FOXP3 to DNA is sufficient to mediate the expression of CD25 or other factors that cooperate with FOXP3 are required to favour the occupancy of *Cd25* promoter by FOXP3 is still unclear.

Accumulating evidence suggests that the transcription factor NF-κB in T lymphocytes is the most relevant target downstream of CD28 biochemical pathways [20]. We have recently demonstrated that the expression of FOXP3 in human CD4^+^CD25**^−^** T lymphocytes can be induced by CD28 unique signals through nuclear translocation of RelA/NF-κB, occupancy of newly identified κB-binding sites on *FOXP3* promoter and FOXP3 trans activation [21], [22]. The expression of FOXP3 in CD28-stimulated CD4^+^CD25**^−^** T lymphocytes correlated with CD25 expression, highlighting a link between FOXP3 and CD25, and a crucial role of RelA for their activation.

Starting from the above reported data, in this article we explored the mechanisms by which FOXP3 positively regulates CD25 expression. We found that the CD28-induced RelA is required to promote FOXP3 binding to DNA at two tandem copies of a non-consensus FOXP3 binding site in the *Cd25* promoter. We also found that the occupancy of both FOXP3 binding sites in conjunction with RelA bound to a κB binding site promotes the transcriptional activation of *Cd25* gene. Our data highlight the need of RelA in the trans activation of *Cd25* gene by FOXP3 and indicate that FOXP3 coordinated binding to the two identified non-consensus binding sites could favour FOXP3 dimerization and activatory function.

## Materials and Methods

### Cell Lines, Antibodies and Reagents

Human peripheral blood mononuclear cells (PBMC) were obtained from exceeding healthy donor buffy coats from the Blood bank of Sapienza University, Rome. PBMC were prepared by centrifugation over Lympholyte-H (Cederlane, Hornby, Canada) gradients. Isolation of human CD4^+^ or CD4^+^CD25**^−^** T cells was performed by human regulatory T cell isolation kit (Miltenyi Biotec, Auburn, CA) according to manufacturer’s instructions. The purity of CD4^+^CD25**^−^** T cell population was confirmed to be >95% by flow cytometry. CD4^+^CD25**^−^** T cells were cultured in RPMI 1640 (Gibco-BRL, Grand Island, NY) supplemented with 2% human serum (Euroclone, UK). Murine L cells (Dap3) and murine L cells expressing human B7.1 (Dap3/B7) were cultured in complete DMEM (Gibco-BRL, Grand Island, NY) supplemented with 50 µg/ml hygromycin B (Sigma Aldrich, St Louis, MO). HEK 293 cells [23], provided by Dr M. Crescenzi (Istituto Superiore di Sanità, Rome, Italy) were cultured in DMEM (Gibco-BRL, Grand Island, NY). Jurkat T cells [24], provided by Dr L. Tuosto (Department of Biology and Biotechnology, Sapienza University, Rome, Italy) were cultured in RPMI 1640 (Gibco-BRL, Grand Island, NY), each containing 10% FCS and penicillin and streptomycin (Sigma Aldrich, St Louis, MO). Anti-FOXP3 (H-190), anti-p65/RelA (C20), anti-HA (Y11), anti-PCNA (PC-10), anti-GAPDH (FL-335) and anti-α tubulin Abs were purchased from Santa Cruz Biotecnology (Santa Cruz, CA). Anti-acetyl-histone H4 was purchased from Upstate Biotechnology (Lake Placid, NY, USA).

### Cell Stimulation, Immunoprecipitation and Immunoblotting

CD4^+^CD25**^−^** T cells were activated either with Dap3 (used as control) or Dap3/B7, as previously described [21]. At the end of incubation, cell were harvested and lysed for 30 min on ice in 1% Nonidet P-40 lysis buffer in the presence of protease and phosphatase inhibitors. Extracts were precleared for 1 h with Protein-A Sepharose and then immunoprecipitated for 2 h with specific Abs pre-adsorbed on Protein-A Sepharose beads (Amersham, UK). Cytoplasmic and nuclear extracts were prepared as previously described [25]. Proteins were resolved by SDS-PAGE and blotted onto nitrocellulose membranes. Blots were incubated with the indicated primary antibodies, extensively washed and after incubation with horseradish peroxidase (HRP)-labeled goat anti-rabbit or goat anti-mouse Abs (Amersham Pharmacia) developed with the enhanced chemiluminescence’s detection system (Amersham Pharmacia).

### Reverse Transcription and Real-time PCR

Total RNA, extracted using TRIzol reagent (Invitrogen), was reverse-transcribed into cDNA by using Moloney murine leukemia virus reverse transcriptase (Invitrogen). FOXP3 and CD25 mRNA expression was determined by the TaqMan method of real-time PCR with glyceraldehyde-3-phosphate dehydrogenase (GAPDH) as endogenous control. TaqMan Universal PCR Master Mix and the FOXP3 primer/probe set (part No. Hs00203958_m1), the CD25 primer/probe set (part No. Hs00907778_m1) and the GAPDH primer/probe set (part No. Hs99999905_m1) were purchased directly from Applied Biosystems. Relative quantification was performed using the comparative C_T_ method as described by Applied Biosystem. All amplifications were conducted in triplicates.

### Cloning of the CD25 Promoter, and Construction of Mutant Constructs

The human *Cd25* promoter containing –546 bp from transcription start site (TSS) was amplified by PCR using CD25 promoter sequence-specific primers from position –546 to +163. The genomic DNA extracted from CD4^+^ T cells of a healthy donor was used as a template. The CD25 promoter amplicon was cloned into the pGL4 basic vector (Promega) to generate the pGL4-CD25 promoter luciferase reporter vector. The FOXP3 and κB binding site mutants were derived from pGL4-CD25 promoter luciferase construct by substituting five nucleotides within the consensus-binding sites by PCR. Primers used to generate the individual constructs are listed in Table S1. The entire sequences of the mutants were verified by DNA sequencing.

### Plasmids, Cell Transfection and Reporter Gene Assays

FOXP3 WT expression plasmid [26] was generously provided by Dr A. Rao (La Jolla Institute for Allergy and Immunology, La Jolla, CA). FOXP3ΔE251 expression plasmid [6] was kindly provided by Dr S.F. Ziegler (Benaroya Research Institute, Seattle, WA). HA-tagged p65/RelA and p50 expression vectors were kindly provided by Dr G. Natoli (Department of Experimental Oncology, European Institute of Oncology, Milan, Italy). HEK 293 cells were transfected as previously described [22]. After 24 h luciferase activity was measured by the dual luciferase assay system (Promega) according to the manufacturer’s instructions. Data were normalized by the activity of Renilla luciferase. Jurkat T cells were transfected by electroporation using 30 µg of total DNA in 400 µl of RPMI 1640 supplemented with 20% FCS. Electroporation was performed in 0.40-cm electroporation cuvettes (Gene Pulser; Bio-Rad, Hercules, CA) at 960 µF and 260 V.

### Bioinformatics

Human *Cd25* promoter sequence was obtained from GenBank with the accession number NG_007403. The FOXP3 binding sites were identified by using the online pattern matching application developed by Dr A. Cabibbo, University of Rome Tor Vergata, freely accessible at: http://immuno.bio.uniroma2.it/cabibbo/toolies/oligo_finder/.

### ChIP and Re-ChIP Assay

Chromatin immunoprecipitation (ChIP) assays were performed as previously described [21]. Briefly, after fixing in 1% formaldehyde, cells were lysed for 5 min in 50 mM Tris (pH 8.0), 20 mM EDTA, 0.1% Nonidet P-40 and 10% glycerol supplemented with proteases inhibitors. Nuclei were resuspended in 50 mM Tris ( pH 8.0), 1% SDS and 5 mM EDTA. Chromatin was sheared by sonication, centrifuged and diluted 10 times in 50 mM Tris (pH 8.0), 0.5% Nonidet P-40, 0.2 M NaCl, 0.5 mM EDTA. After preclearing with a 50% suspension of salmon sperm-saturated protein A, lysates were incubated at 4°C overnight with the indicated antibodies. Immune complexes were collected with sperm-saturated protein A, washed three times with high-salt buffer (20 mM Tris (pH 8.0), 0.1% SDS, 1% Nonidet P-40, 2 mM EDTA, 500 mM NaCl) and five times with 1 × Tris/EDTA (TE). Immune complexes were extracted in 1 × TE containing 1% SDS, and protein–DNA cross-links were reverted by heating at 65°C overnight. DNA was extracted by phenol–chloroform and about 1/20 of the immunoprecipitated DNA was used in each PCR. For precipitation Abs against p65/RelA, FOXP3 and acetyl-histone H4 were used. The primers used were as follows: CD25, 5′- CTGGGTGAGACCACTGCCAAG -3′ and 5′-CCTCTTTTTGGCATCGCGCCG -3′. Re-ChIP assays utilized a similar protocol, except that the primary immunocomplex obtained with the first antibody was eluted by 10 mM dithiothreitol with agitation at 37°C for 30 min. The eluate was diluted 50 times with buffer (20 mM Tris-HCl (pH 8.0), 150 mM NaCl, 2 mM EDTA and 1% Triton X-100/ and immunoprecipitated with the second antibodies.

### FOXP3 siRNA Experiments

FOXP3 siRNA (primers HSS121456-57) were purchased from Invitrogen (Stealth Select RNAi). The primers used were as follow: HSS121456 5′-CCACAACAUGGACUACUUCAAGUUC-3′, and HSS121457 5′- GCCACAUUUCAUGCACCAGCUCUCA-3′ corresponding to positions 1163–1187, and 485–509 relative to the start codon, respectively. The negative control was a scrambled sequence with no homology to human, mouse or rat genomes. To transfect the CD4^+^CD25**^−^** T cells, 200 pmol of siRNA was mixed with 100 µl of the human T-cell Nucleofector solution. Cell suspension was immediately electroporated by the Nucleofector II instrument (Amaxa biosystems, Gaithersburg, MD). The transfected CD4^+^CD25**^−^** T cells were rested in culture medium for 24 h before being stimulated with adherent Dap3/B7.

### Electrophoretic Mobility Shift Assay

Single-stranded oligonucleotides containing the putative FOXP3 binding sites were annealed with their complementary strands and purified for use as probes in electrophoretic mobility-shift assays (EMSA). The following oligonucleotide sequence (one strand with putative binding sites underlined) were end-labeled with γ^32^P-ATP using T4 polynucleotide kinase in accordance with manufacturers’ instructions:

CD25, 5′- ACTTGAAAAAAAAAACCTGGTTTGAAAAATTA- 3′;

CD25 1mut, 5′- ACTTGCCCGGAAAAACCTGGTTTGAAAAATTA- 3′.

CD25 2mut, 5′- ACTTGCCCGGAAAAACCTGGTTTGCCCGGTTA- 3′.

The oligonucleotide sequence of *Cd25* gene encompassing the sequence −469 −438 was also used in EMSA competition assays as control.

Nuclear extracts of Jurkat T cells, prepared as previously described [25], were controlled for equal protein content by a protein assay, as described by the manufacturer (Bio-Rad).

Binding reactions were performed at room temperature for 20 minutes using 5 µg of nuclear extracts and approximately 10,000–20,000 c.p.m. (<0.1–0.5 ng) of ^32^P-end labeled probes in 20 µl. The final concentration of components of the binding buffer for all EMSA experiments were: 12 mM HEPES (pH 7.5), 100 mM KCl, 1 mM DTT, 1 mM EDTA, 12% glycerol and 20 µg/ml poly(dI)-poly(dC). Unlabeled double-stranded competitor or control oligonucleotides were preincubated with cell extracts 20 min prior to addition of the probe. DNA-protein complexes were separated from free probe by electrophoresis in a 5% polyacrylamide, TBE gel containing 1% glycerol. After electrophoresis, dried gel was exposed at −80°C with intensifying screen, according to the intensity of the radioactive signals.

## Results

### CD28 Signals Promote the Physical Interaction of RelA/NF-κB and FOXP3 on *Cd25* Promoter and Histone Acetylation

**Figure 1:**
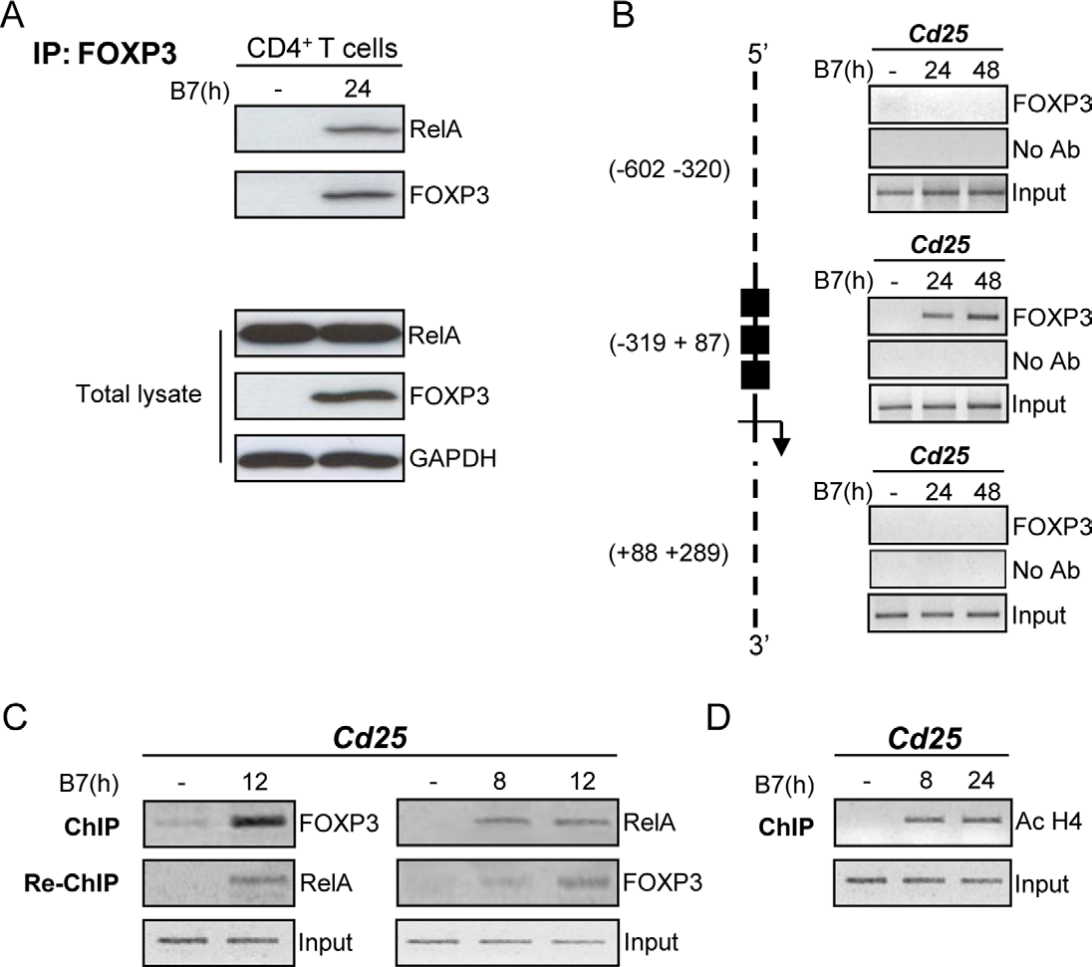
RelA and FOXP3 interact with each other on *Cd25* promoter in CD28-stimulated CD4^+^CD25^−^ **T cells.** CD4^+^ T cells or CD4^+^CD25**^−^** T cells were stimulated with adherent Dap3/B7 cells for the indicated times. (**A**) Endogenous FOXP3 was immunoprecipitated from equal amounts of total lysates of stimulated or unstimulated CD4^+^ T cells and the association with RelA was analysed by Western blotting (upper panels). The expression of RelA, FOXP3 and GAPDH was evaluated by Western blotting (lower panels). (**B**) ChIP assays were performed by using anti-FOXP3 Ab on stimulated or unstimulated CD4^+^CD25**^−^** T cells. Scheme of the 5′UTR region of *Cd25* gene with the position of FOXP3 binding region is reported. (**C**) Cells were then analysed by ChIP and Re-ChIP assays by using anti-RelA or anti-FOXP3 Abs or (**D**) by ChIP for acetyl-histone H4 binding to the *Cd25* promoter. (**B–D**) Immunoprecipitated DNA was analysed by PCR with *Cd25* promoter-specific primers. The Input represents PCR amplification of the total sample, which was not subjected to any precipitation. Shown results are representative of three independent experiments.

Since the transcription of *Cd25* gene is mainly dependent upon NF-κB nuclear translocation and binding to κB sites [17], we asked whether RelA could interact with FOXP3 and the complex RelA-FOXP3 could mediate the activation of CD25 expression. Therefore, according to the evidence that FOXP3 ectopically expressed in 293T cells interacted with endogenous RelA [10], we first verified the presence of RelA-FOXP3 complex in human CD4^+^ T cells stimulated for 24 h by the interaction of CD28 with B7, expressed on Dap3 cells (Dap3/B7), by immunoprecipitation. FOXP3 was immunoprecipitated by anti-FOXP3 antibody and Western blotting with anti-RelA revealed that FOXP3 physically associated with RelA (Figure 1A), evidencing that FOXP3 either endogenous or overexpressed interacts with RelA. Secondly, we used ChIP assays to verify whether the complex RelA-FOXP3 binds to *Cd25* promoter. Initially, chromatin of CD4^+^CD25**^−^** T cells stimulated for 24 and 48 h by B7 was precipitated with anti-FOXP3 to define the *Cd25* chromatin region bound by FOXP3. To this aim, the regions encompassing the nucleotides −602 −320, −319 +87 and +88 +289 of *Cd25* promoter were amplified with specific primers. The results of Figure 1B show that the only region where FOXP3 was recruited for all of the observation time was included between –319 and +87 nucleotides. Furthermore, this region was analysed for the co-presence of both FOXP3 and RelA at earlier time points, Figure 1C. Interestingly, RelA occupied the region –319 +87 at 8 h and both FOXP3 and RelA were recruited at 12 h after CD28 activation. Since signals from CD28 activated histone acetyltransferase (HAT) enzymes that may influence gene expression through epigenetic mechanisms [27], the histone acetylation of the region bound by FOXP3 and RelA was also analysed [8]. Figure 1D shows the acetylation of histone H4 molecule after 8 h and 24 h from CD28 stimulation. Finally, Re-ChIP was used to establish whether the two transcription factors were present in a transcriptional complex at the –319 +87 DNA sequence. Both FOXP3:DNA and RelA:DNA complexes, obtained from the first immunoprecipitation with anti-FOXP3 and anti-RelA, respectively, were additionally immunoprecipitated with anti-RelA and anti-FOXP3, respectively. The second row of Figure 1C shows that in CD4^+^CD25**^−^** T cells at 12 h from CD28 stimulation RelA and FOXP3 physically associate on *Cd25* promoter. Taken together, these findings indicate that the upregulation of CD25 expression in CD4^+^CD25**^−^** T cells by CD28 signals could be dependent on the complex RelA-FOXP3 associated with *Cd25* promoter.

### Characterization of the FOXP3 Binding Sites on *Cd25* Promoter

**Figure 2:**
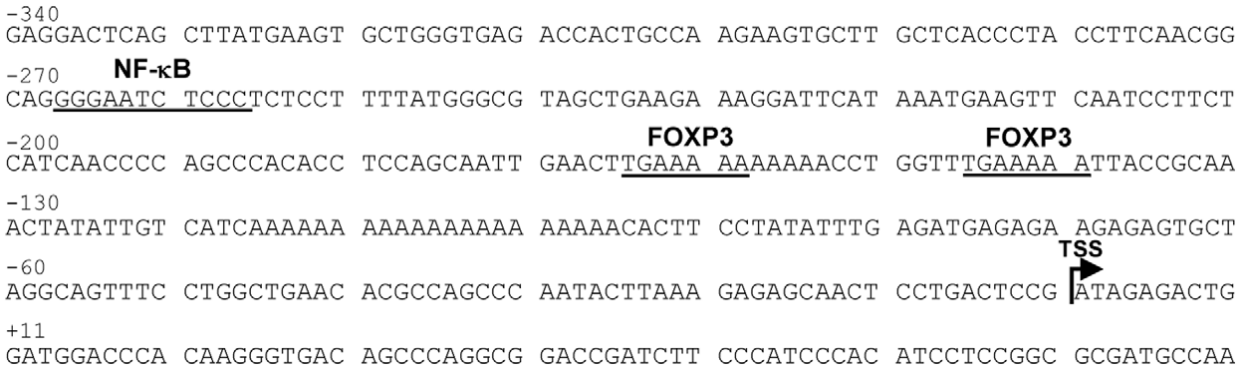
Putative FOXP3 binding sites in *Cd25* promoter. The partial human *Cd25* promoter sequence (–340 +80) is reported (GenBank accession n° NG_007403). The TSS is indicated by broken arrow. The underlined regions represent the κB binding site (–267) and the two putative FOXP3 binding sites (–165 and –146).

**Figure 3:**
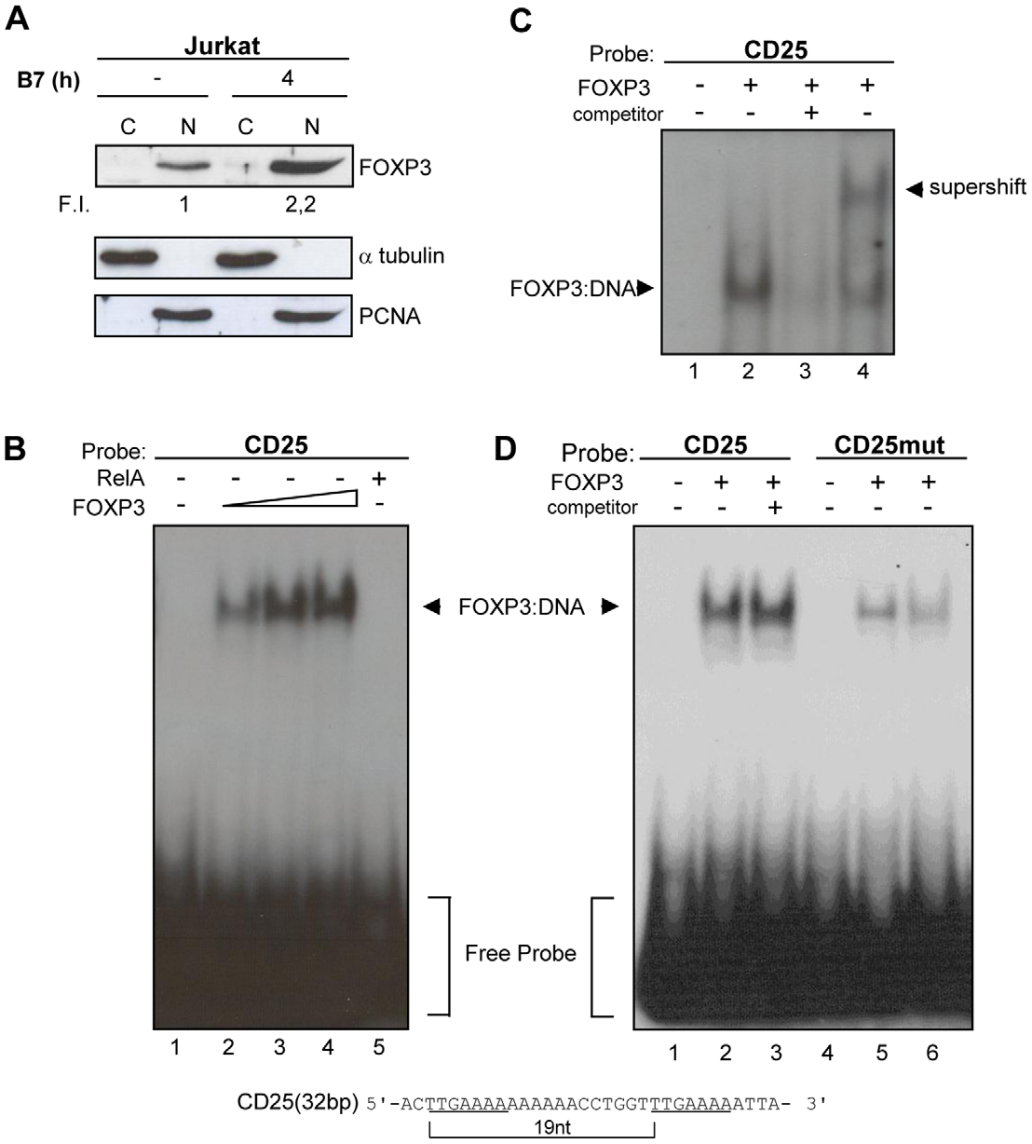
FOXP3 binds to oligonucleotides containing two tandem copies of putative FOXP3 binding site in *Cd25* promoter. Jurkat T cells were transfected with FOXP3 expression vector and stimulated with adherent Dap3/B7 cells for 4 h. (**A**) Anti-FOXP3 Western blotting analysis of both cytosolic and nuclear extracts of Jurkat T cells. The blots were reprobed with anti-α tubulin (cytosol) or anti-PCNA (nucleus) Abs for equal loading of proteins. Fold of induction (F.I.) over the basal level are indicated. The data represent at least three independent experiments. (**B**) Nuclear protein extracts from Jurkat T cells, transfected with increased concentration (5, 10, 20 µg) of FOXP3 (lane 2–3–4) or RelA (10 µg, lane 5) expression vectors and activated, were incubated with labelled probe as indicated. Protein:DNA complexes and free probe are indicated at left (by arrowhead in one case and a square bracket in the other). (**C**) Nuclear protein extracts from activated Jurkat T cells transfected with 10 µg of FOXP3 was incubated with unlabeled oligonucleotide competitor excess (50-fold, lane 3) or with anti-FOXP3 polyclonal Ab to determine a supershift reaction (by arrowhead at right, lane 4). (**D**) Nuclear protein extracts, prepared as above described (panel C), were incubated with labelled CD25 probe in presence (lane 3) or absence (lane 2) of unlabelled oligonucleotide competitor encompassing the *Cd25* sequence −469 −438 or with labelled CD25 probes mutated at −165 (lane 5) or at −165 and −146 (lane 6) FOXP3 binding sites.

Although sequence analysis of *Cd25* promoter has confirmed the presence of a major transcription initiation κB site at position –267 which can drive CD25 expression [17], the presence of FOXP3 binding motifs mediating FOXP3 dependent trans activation of *Cd25* gene has not yet been identified. Sequence analysis of region –340 +80 of *Cd25* promoter (Figure 2) has identified at positions –165 and –146 two tandem copies of the sequence 5′-TGAAAAA-3′ that matches the complement of FOXP3 binding sequence 5′-TGTTTCA-3′ in the *Il2* promoter except for the substitution at position 2 of G with T [9]. The two identified sequences were separated at 5′ ends by 19 nucleotides, a distance very close to two helical repeats in the B-form DNA. Since it has been demonstrated *in vitro* that FOXP3 FKH domain can simultaneously bind two distinct FOXP3 binding sites separated by a flexible 19-base single-stranded DNA linker [7], [27], we hypothesized that the sequence 5′- TGAAAAA -3′ could represent a putative FOXP3 binding site in *Cd25* promoter. To explore this possibility, we tested in EMSA assays whether nuclear protein extracts of Jurkat T cells, transfected with FOXP3 expression vector and activated by Dap3/B7, could bind oligonucleotides of 32 base-pairs (bp), encompassing the two putative FOXP3 binding sites. As control, nuclear protein extracts of Jurkat T cells transfected with RelA expression vectors were used. The results of immunoblots (Figure 3A), performed to analyse the nuclear levels of exogenous FOXP3 proteins in both stimulated and unstimulated cells, show that CD28 signals induced an increased expression of FOXP3 protein levels exclusively localized in the nuclei. Since the activation of CD28 pathways by B7 leads to the preferential nuclear translocation of RelA [20], we also verified whether the nuclear presence of RelA and its binding to DNA could be responsible of the increase of FOXP3 nuclear levels. Jurkat T cells were cotransfected with either RelA and FOXP3 or with a RelA mutant, RelA YA ED, unable to bind canonical κB sites, [28] and FOXP3. The immunoblots in the Figure S1 show that the nuclear presence of exogenous RelA resulted in a significant increase of FOXP3 levels (PanelA) while the presence of RelA YA ED did not modify FOXP3 expression (Panel B). Panels B, C and D of Figure 3 show EMSA results obtained with nuclear extracts of CD28-stimulated FOXP3 transfected Jurkat T cells. The presence of a retarded band that increased proportionally to the increase of FOXP3 expression in nuclear extracts (lane 2,3,4 of Figure 3B) and the absence of probe occupancy in nuclear extracts over-expressing RelA (lane 5), suggested a binding between FOXP3 and DNA. The FOXP3 binding specificity was determined by either supershift analysis or competition experiments. The evidence that the complexes were supershifted using anti-FOXP3 antibody (lane 4 of Panel C), and that 50 fold excess of unlabelled nucleotides totally abrogated the retarded band (lane 3 of Panel C) support the specificity of the interaction between FOXP3 and the probe. EMSA assays were also repeated with probes mutated in one or two FOXP3 binding sites (CD25 1mut and CD25 2mut as described in Materials and Methods). As shown in Panel D, the progressive mutation of one (lane 5) or two (lane 6) FOXP3 binding sites profoundly decreased the retarded band. The addition of the unlabelled nucleotides encompassing *Cd25* gene sequence −469 −438 (50 fold) in lane 3 of Panel D did not modify the retarded band. Altogether, these data indicate that we have identified a sequence on *Cd25* promoter, characterized by two binding sites both required for optimal DNA-binding by FOXP3, that represent a FOXP3 binding region.

### RelA and FOXP3 Synergize to Trans Activate *Cd25* Promoter

**Figure 4:**
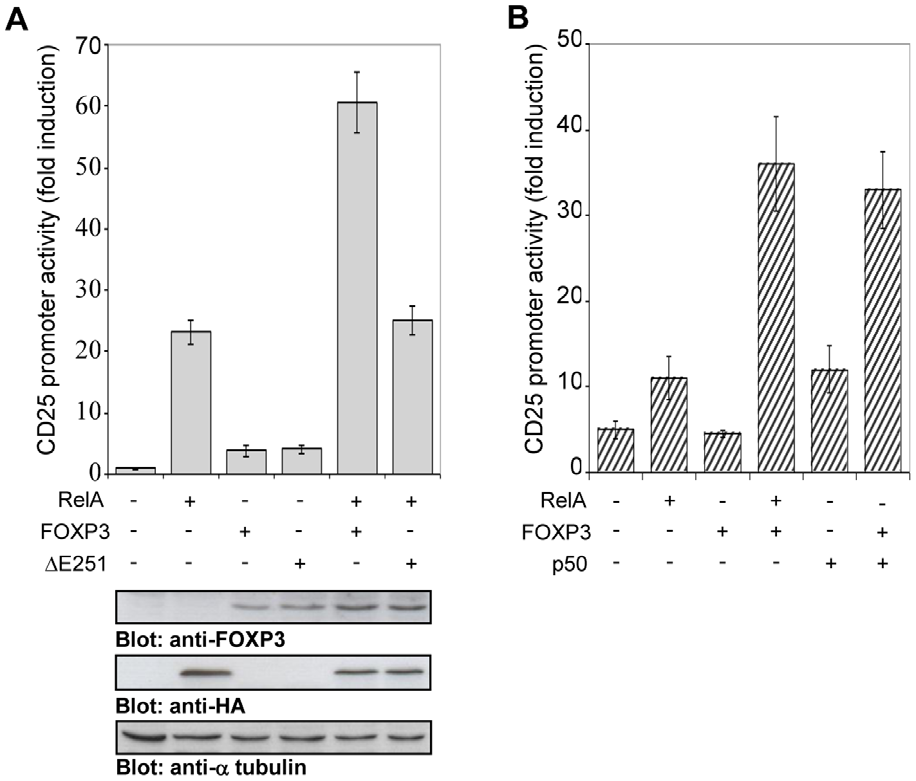
FOXP3 and RelA synergize to trans activate *Cd25* promoter in HEK 293 cells. HEK 293 cells were transfected with CD25 promoter luciferase reporter vectors and cotransfected with HA-tagged RelA or FOXP3 or FOXP3ΔE251 (**A**) or p50 (**B**) expression plasmids, where indicated. Data are the mean ± SD of luciferase light units normalized for Renilla luciferase of the same sample. Results are representative of five independent experiments performed in triplicate. An aliquot of each sample (A, lower panel) was analysed by immunoblotting with anti-FOXP3, anti-HA and anti-α tubulin Abs.

The evidence that FOXP3 and RelA bound directly to the *Cd25* promoter in the same complex supports the possibility that FOXP3 is capable of functioning as a co-activator of *Cd25* gene in association with RelA. However, while RelA has been described to trans activate *Cd25* promoter, a direct effect of FOXP3 is not clear. To examine the functional responsiveness of the *Cd25* promoter to FOXP3, a CD25 promoter-luciferase construct was generated. Expression vectors encoding RelA, FOXP3 and FOXP3 mutant ΔE251 (FOXP3ΔE251), an IPEX mutation in the leucin zipper domain that abrogates the FOXP3 ability to homodimerize [6], have been used to analyse the trans activation of the construct. HEK 293 cells were cotransfected with the CD25 construct and RelA or FOXP3 or FOXP3ΔE251, or RelA and FOXP3 or RelA and FOXP3ΔE251expression vectors. After 24 h, luciferase reporter gene activity was measured such as RelA and FOXP3 protein levels (Figure 4). Although RelA shows clear activatory properties (Figure 4A), FOXP3 vectors did not affect the activity of the CD25 construct. However, co-expression of RelA and FOXP3 resulted in a synergic effect on promoter activity, whereas the mutant FOXP3ΔE251was not transcriptionally functional [29]. The effect of the NF-κB p50-p65 heterodimeric complex, the main form of NF-κB regulating CD25 expression [17], on the activation of the CD25 construct in the presence or absence of FOXP3 was also tested. The endogenous NF-κB trans activation was induced by overexpression of p50. The results of Figure 4B show that the activity of FOXP3 on CD25 construct is equally dependent on the overexpression of either p50 or p65 subunits of NF-κB.

It has been reported that the physical association of FOXP3 with Rel family transcription factors blocked their ability to induce the endogenous expression of their target genes [10]. However, many other evidences sustain the view that the transcriptional activity of FOXP3, as a repressor or an activator, is dependent on the target gene involved. To verify whether FOXP3 could both activate *Cd25* and inhibit *Il2* promoter activity when co-expressed with RelA in HEK 293, we repeated the experiments of Figure 4A by substituting CD25 promoter construct with IL-2 promoter construct. The results of Figure S2 clearly show that FOXP3 efficiently suppressed *Il2* gene expression. Less evident suppression of luciferase activity was obtained with the FOXP3ΔE251 expression vector, as previously observed [30].

**Figure 5:**
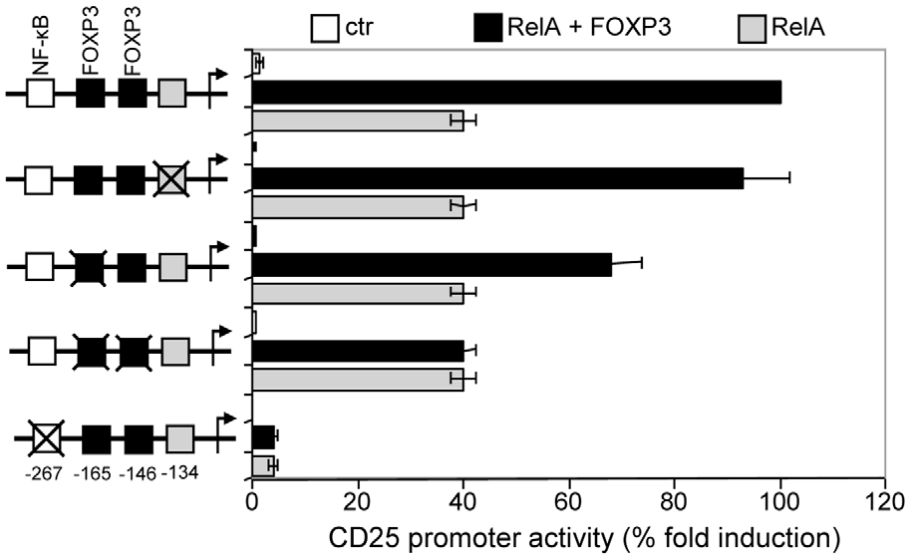
The binding of FOXP3 to two non-consensus binding sites arrayed in tandem concurs to the trans activation of *Cd25* promoter. Four different constructs of human *Cd25* promoter region were generated by mutation in a site-directed manner as indicated. Both wild-type and mutated luciferase reporter vectors were transfected into HEK 293 cells. Where indicated, the cells were cotransfected with HA-tagged RelA and FOXP3 expression plasmids. Data are the mean ± SD of luciferase light units normalized for Renilla luciferase of the same sample. Effect of mutagenesis is shown as percentage relative to wild-type construct, cotransfected with HA-tagged RelA and FOXP3, and arbitrarily set to 100%.

Furthermore, the functional role of FOXP3 and κB binding sites in *Cd25* promoter was analysed by site directed mutagenesis of these sites. Four different constructs were generated as described in Supporting Information Table S1, including the sequence 5′-GCAAACT-3′ at position –134 that presented a correspondence of the first six nucleotides with a FOXP3 consensus site. The mutant constructs were used in luciferase experiments in the presence of RelA or RelA and FOXP3. The results of Figure 5, where the levels of activation mediated by RelA and FOXP3 were put in relation to those mediated by RelA alone in the culture, show that the mutation of –134 sequence did not modify the CD25 reporter gene activation. However, when the two FOXP3 binding sites were progressively mutated, a significant decrease of the reporter gene activities was obtained. Indeed mutations of both –165 and –146 sequences brought the level of activation to that obtained by RelA alone in culture, suggesting that both binding sites on *Cd25* promoter can act co-ordinately to exert trans activation of *Cd25* promoter. However, the loss of the luciferase activity of CD25 promoter was only obtained by mutation of κB site at position –267, suggesting that FOXP3-dependent activation of the *Cd25* promoter is first dependent on the RelA transcription factor complex. Therefore, RelA is the principal activator of *Cd25* promoter and the binding to its κB site is required to mediate the FOXP3 activatory function. Collectively, these data strongly suggest that the positive transcriptional activity of FOXP3 on *Cd25* promoter could be dependent on the binding of RelA to its κB site and on the interaction between FOXP3 and RelA that could favour FOXP3 binding to the two tandem copies of the sequence 5′-TGAAAAA-3′ with consequent trans activation of *Cd25* gene.

### Knockdown of FOXP3 in CD28-activated T Cells Inhibits CD25 Expression

**Figure 6:**
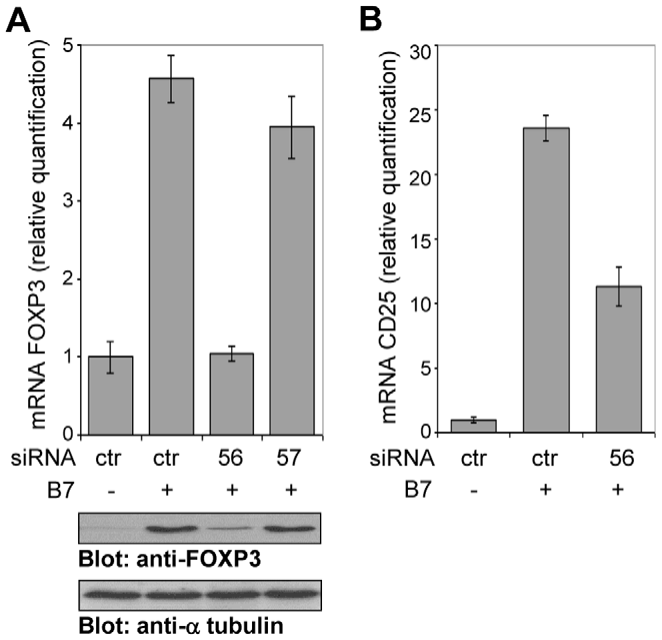
siRNA-mediated knockdown of FOXP3 inhibits *Cd25* gene expression in CD28-activated CD4^+^CD25^−^ **T cells.** Human CD4^+^CD25**^−^** T cells were transfected with FOXP3 siRNA (56, 57) or control siRNA (ctr) and activated with adherent Dap3/B7. (**A**) FOXP3 mRNA and protein levels were measured by real-time PCR (upper panel) and immunoblots (lower panels) after 24 h of stimulation. Data are shown from one of three similar experiments. (**B**) CD25 mRNA was measured by real-time PCR after 24 h. Data are representative of four independent experiments.

Since FOXP3 enhances CD25 expression induced by RelA, the knockdown of FOXP3 in CD4^+^CD25**^−^** T cells stimulated by CD28 would be expected to reduce CD25 expression. To address this possibility anti-CD28 stimulated CD4^+^CD25**^−^** T cells were transfected with two different FOXP3 siRNA (56 and 57) and a control siRNA. The knockdown of FOXP3 was determined by real-time PCR and Western blotting after 24 h (Figure 6A). The effect of primer 56, that significantly decreased FOXP3 expression, and control siRNA on CD25 expression was analysed in CD4^+^CD25**^−^** T cells stimulated by CD28. The results of Figure 6B show that 56 siRNA induced 40% inhibition of CD25 mRNA synthesis measured after 24 h. An inhibitory effect of the knockdown of FOXP3 on CD25 expression measured by FACS at the single cell level as MFI after 48 h of culture was also observed (data not shown). Altogether these data confirm that RelA and FOXP3 synergize to trans activate *Cd25* promoter and are consistent with FOXP3 serving as a co-activator of CD25 expression.

## Discussion

Considerable evidence supports the prediction that CD25 is directly regulated by FOXP3. However, given that CD25 is normally upregulated in all activated T cells, regardless of whether they express FOXP3, and the difficulty of analysing *in vitro* signal transduction pathways that specifically target FOXP3 to *Cd25* promoter, this issue has still to be definitively demonstrated. Here we have analysed the mechanisms that could account for a direct regulation of CD25 expression by FOXP3. Our data are in agreement with a model where both RelA and FOXP3 serve as co-activators of *Cd25* gene expression. Indeed we show that a cooperative binding between RelA and FOXP3 on a regulatory region of *Cd25* promoter, where κB and two non-consensus FOXP3 binding sites are present, allows FOXP3 to upregulate CD25 expression.

The first observation was the recruitment of both RelA and FOXP3 on *Cd25* promoter and their physical association that supports a *scenario* where CD28 costimulation may influence the interaction of FOXP3 with RelA in a DNA sequence-dependent fashion. Although a physical interaction between FOXP3 and RelA has been described [10], DNA binding of the complex has not been determined. Therefore, our data are, to our knowledge, the first direct evidence of an interaction between endogenous FOXP3 and RelA at *Cd25* promoter. The interaction of FOXP3 with DNA and with other transcription factors represents two important features of its transcriptional activity. There is structural evidence for an interaction between NFAT and FOXP3 [9] and biochemical and functional evidence for a cooperative interaction between FOXP3 and either NFAT or Runx1 at the *Il2* promoter [9], [11]. Both protein-protein and DNA-binding domain interactions have been deeply analysed, suggesting the formation of a tripartite interaction that could repress *Il2* gene expression [31]. Similarly, the tripartite interaction has been supposed to regulate the increase of *Cd25* expression in Treg. Although the conditions that could regulate the interaction among these three transcription factors at *Cd25* promoter are less known, our data sustain NF-κB as a further component of a larger complex that could regulate *Cd25* gene expression in Treg.

Previous studies have shown that a promoter/enhancer region of *Cd25* gene, spanning the nucleotides −276 −64 relative to the major transcription initiation site, controlled at least in part CD25 expression in T cells [32]. This critical region contains an NF-κB binding site at position −267 −257 [17] and the occupancy of this site by various Rel/NF-κB complex induced by CD28 co-stimulatory signals is required to trans activate *Cd*25 in conventional CD4^+^ T cells [33]. FOXP3 has been also described as an activator of CD25 expression [18], [19] but the direct binding of this factor to CD25 DNA has not yet been revealed. Here we present the evidence that FOXP3 directly binds to *Cd25* gene and activates its transcription. Indeed we have identified two non-consensus FOXP3-binding sites that bind FOXP3 down-stream of the known κB-binding site at positions –165 and –146. Moreover, by using a reporter construct composed of the promoter/enhancer region of *Cd25* gene we have not only confirmed the role of NF-κB factors as potent activators of *Cd25* promoter, but we have also confirmed FOXP3 as a direct activator of *Cd25* gene. However, the capacity of both RelA and FOXP3 to regulate *Cd25* gene raises a question: how to reconcile the role of RelA and FOXP3 in the regulation of CD25 expression in Treg. Our data, demonstrating that the simple binding of FOXP3 to *Cd25* promoter is not sufficient to activate CD25 expression, support the hypothesis that FOXP3 could acquire activatory function in a cooperative complex with RelA, and both RelA and FOXP3 function as direct activator of *Cd25* promoter in Treg. The reduction of both FOXP3 and CD25 expression in Treg generated in mice deficient of NF-κB activatory proteins strongly supports this possibility [34]. Therefore the complex FOXP3-RelA-DNA could represent a minimal complex required to stimulate *Cd25* gene in Treg as the complex FOXP3-NFAT-DNA has been described as a minimal complex required to inhibit *Il2* gene in the same cells [8], [9] although with different requirements for the interaction FOXP3-RelA-DNA and FOXP3-NFAT-DNA. Indeed, FOXP3 and RelA interact on sites that are separated in *Cd25* promoter, while FOXP3 and NFAT interact on ‘composite’ DNA elements with adjacent binding sites for FOXP3 and NFAT in *Il2* promoter [9]. However, the presence of NFAT binding site located at approximately –650 and –585 upstream of the promoter region in the mouse *Cd25* gene, suggests that additional transcription factors could be linked to FOXP3 and assemble complex larger than FOXP3-RelA-DNA to upregulate CD25 expression.

It is widely accepted the CD4^+^CD25^+^FOXP3^+^ T cell express higher level of CD25 on membranes respect to CD4^+^CD25^+^FOXP3**^−^** conventional T cells. In accordance with this, we observed that in CD4^+^CD25**^−^** T cells stimulated with anti-CD28 mAb the levels of CD25 molecules were significantly higher in FOXP3^+^ than in FOXP3**^−^** T cells (data not shown). This suggests that the cooperative binding of FOXP3 and RelA on *Cd25* target gene could result in a synergistic effect. The marked decrease in *Cd25* mRNA in CD28 stimulated CD4^+^CD25^+^FOXP3**^−^** T cells, where the knockdown of FOXP3 has been induced by RNAi, supports this hypothesis. The critical role of RelA for FOXP3 enhancement of target genes has been demonstrated for HIV-1 long terminal repeat (LTR) [35]. The authors have shown that FOXP3 enhances HIV-1 LTR *via* specific NF-κB binding sequences in an NF-κB-dependent fashion. However, how FOXP3 influences *Cd25* gene expression remains an open question. Indeed, the increased histone acetylation of the region where RelA-FOXP3-DNA complex were assembled, could suggest that FOXP3 recruits histone acetyltransferase enzymes (HAT), which are powerful co-activators of transcription. Furthermore, although it has been described that endogenous FOXP3 consistently associated with HAT such as Tip60 [12] or p300 [14], whether FOXP3 can recruits histone acetyltransferase to gene promoters in T cells is not known. In addition, DNA binding of RelA could also mediate the recruitment of p300 [36] and consequently p300 may act as a scaffold for the assembly of FOXP3 through modulation of FOXP3 acetylation [14]. Our evidence that exogenous FOXP3 failed to trans activate *Cd25* promoter in the absence of κB site occupancy by RelA, supports a model whereby RelA binding to κB sites is required to modulate FOXP3 transcriptional function. The evidence that in a subset of RelA-dependent genes, without the binding of RelA, NF- κB target promoters cannot be bound by many other transcription factors, supports this possibility [37].

It has been proposed that the degenerate nature of FOXP3 binding sites *in vivo* [38], [39] may reflect the contributions of additional co-factors at specific loci [26]. Therefore large-scale ‘ChIP-chip’ assay have been used to identify DNA elements likely to bind FOXP3 *in vivo*, either alone or in complex with transcriptional partners [38], [39]. FOXP3 binding *in vitro* has been observed only on consensus sites, and EMSA experiments confirm that both the DNA-binding FKH domain and intact leucin-zipper domain, which mediates homo-multimerization of FOXP3 are required for DNA binding [26]. Here we have described a FOXP3 binding region, characterized by two non-consensus FKH sites separated at 5′ends by 19 nucleotides, that allows the binding and stabilization of FOXP3 to DNA. Indeed, although the identified FOXP3 target sequences are not consistent with a FKH consensus motif [26], [40] these sites could become permissive *in vitro* to FOXP3 binding at least in two distinct modes: through post-translational modifications of FOXP3 induced by CD28 signals in Jurkat T cells and through the distinctive preference of FOXP3 for tandem sequences in DNA.

Our data also highlight the importance of dimer formation for transcriptional regulation by FOXP3 of target genes. Indeed it has been found that FOXP3 mutant ΔE251 [6] poorly associated with the *Il2* promoter in human T cells and was less efficient at repressing IL-2 transcription [30]. Here FOXP3ΔE251failed to upregulate CD25 expression induced by RelA. Altogether these results suggest that the homodimerization of FOXP3 is crucial for the binding of FOXP3 to DNA but is not implicated in mediating inhibitory or activatory functions.

In conclusion, we have provided novel information on the mechanisms by which RelA and FOXP3 cooperate to mediate transcriptional regulation of target genes and identified a region on *Cd25* promoter where FOXP3 dimer could bridge intramolecularly two DNA sites and trans activate *Cd25* promoter.

## Supporting Information

Figure S1 **RelA upregulates nuclear levels of FOXP3 protein.** For a better evaluation of RelA role and activity, Jurkat T cells were transfected with almost undetectable amounts of FOXP3 expression vector (5 µg). Western blots were performed on cytoplasmic and nuclear extracts. (A) Jurkat T cells were transfected for 24 h with HA-tagged RelA or FOXP3 expression vector alone or in combination. Each sample was analyzed by immunoblotting with anti-FOXP3 and anti-RelA Abs. The blots were reprobed with anti-α tubulin (cytosol) or anti-PCNA (nucleus) Abs for equal loading proteins. (B) Jurkat T cells were transfected for 24 h with HA-tagged RelA or FOXP3 or RelA YA ED expression vector alone or in combination. Each sample was analyzed by immunoblotting with anti-FOXP3 and anti-RelA Abs. The blots were reprobed with anti-α tubulin Ab to verify equal loading of proteins. Fold of induction (F.I.) over the basal level are indicated. The data represent at least three independent experiments.
(TIF)

Figure S2 **FOXP3 efficiently suppressed** ***Il2*** **gene expression in HEK 293 cells.** HEK 293 cells were transfected with IL2 luciferase reporter vector (kindly provided by Dr J.F. Peyron (Facultè de Medicine Pasteur, Nice) and cotransfected with HA-tagged RelA or FOXP3 or FOXP3ΔE251 expression plasmids where indicated. Results given are the mean ± SD of luciferase light units normalized for Renilla luciferase of the same sample. Results are representative of five independent experiments performed in triplicate.
(TIF)

Table S1 **Wild type FOXP3 binding sites sequences and mutated primers employed.** Primers CD25 introduced a restriction enzyme recognition site for *KpnI* or *XhoI*. The primers used for mutational analysis are also shown. In the upper lane, FOXP3 and κB binding sites are evidenced in grey and indicated by position. The primers used for mutational analysis are shown in the lower lane and the underlined letters denote mutated nucleotides. FOXP3^2/3^ mutant was obtained by site-directed mutagenesis using FOXP3^2^ and FOXP3^3^ primers.
(TIF)
