# Supplementary figures and images for: Forkhead Transcription Factor FOXP3 Upregulates CD25 Expression through Cooperation with RelA/NF-κB
Source: PLoS One. 2012 Oct 29;7(10):e48303. doi: 10.1371/journal.pone.0048303 (PMC3483148; doi:10.1371/journal.pone.0048303)

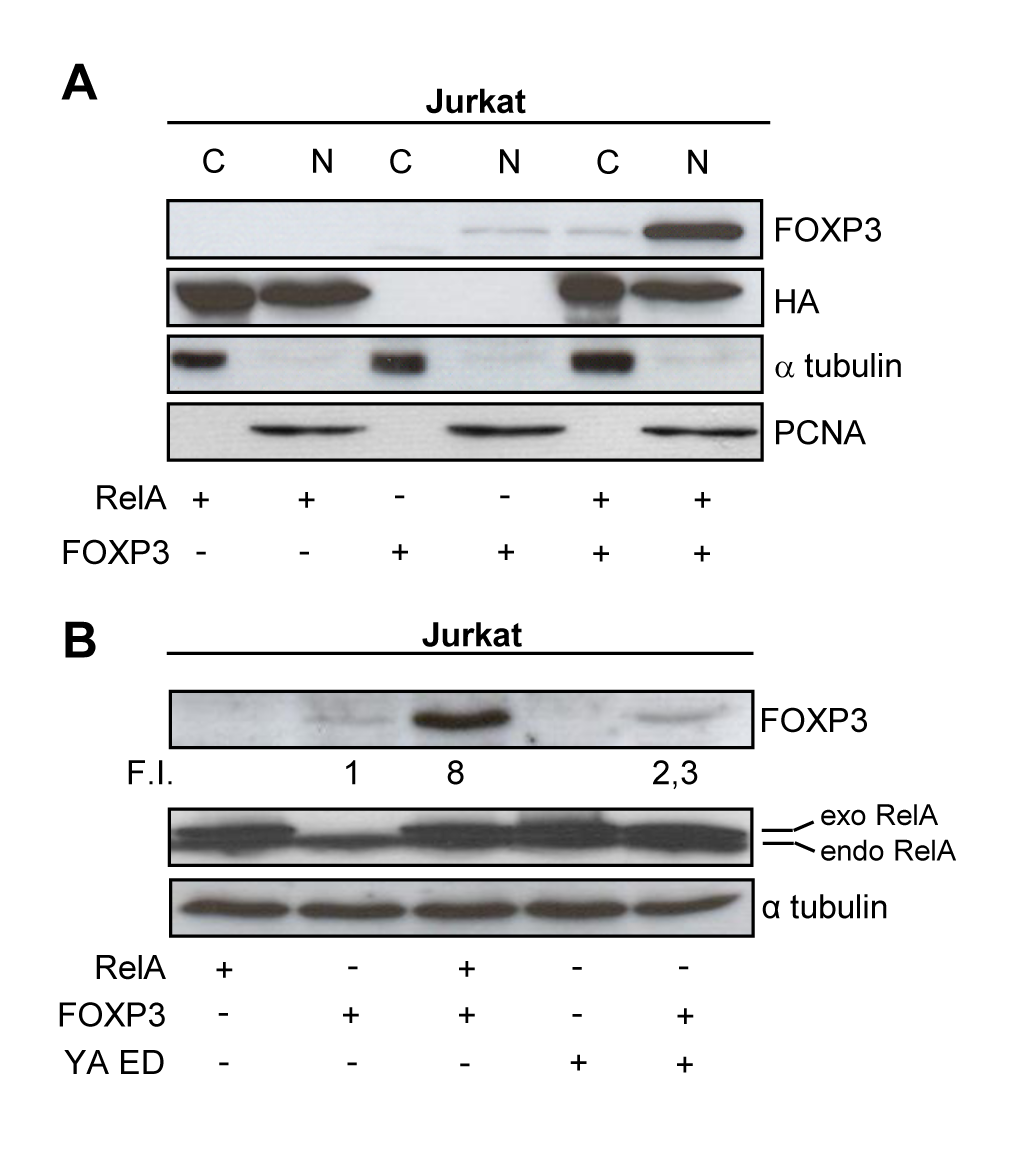

Supplement: Figure S1 — RelA upregulates nuclear levels of FOXP3 protein. For a better evaluation of RelA role and activity, Jurkat T cells were transfected with almost undetectable amounts of FOXP3 expression vector (5 µg). Western blots were performed on cytoplasmic and nuclear extracts. (A) Jurkat T cells were transfected for 24 h with HA-tagged RelA or FOXP3 expression vector alone or in combination. Each sample was analyzed by immunoblotting with anti-FOXP3 and anti-RelA Abs. The blots were reprobed with anti-α tubulin (cytosol) or anti-PCNA (nucleus) Abs for equal loading proteins. (B) Jurkat T cells were transfected for 24 h with HA-tagged RelA or FOXP3 or RelA YA ED expression vector alone or in combination. Each sample was analyzed by immunoblotting with anti-FOXP3 and anti-RelA Abs. The blots were reprobed with anti-α tubulin Ab to verify equal loading of proteins. Fold of induction (F.I.) over the basal level are indicated. The data represent at least three independent experiments. (TIF) [file pone.0048303.s001.tif]

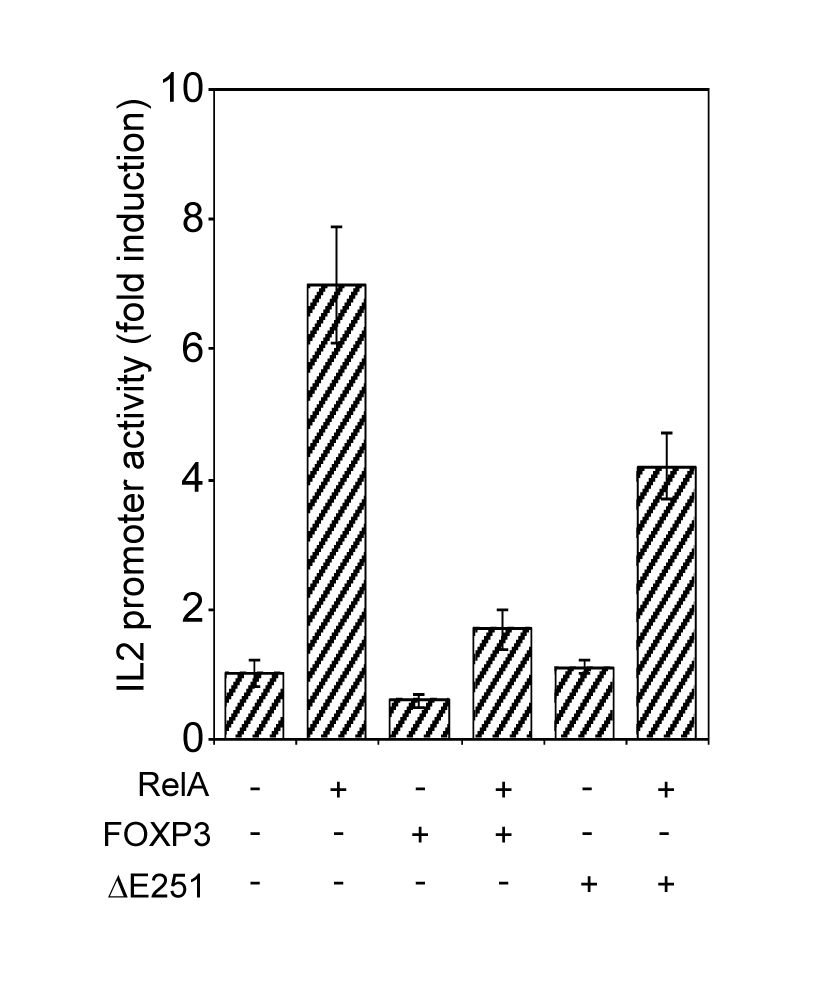

Supplement: Figure S2 — FOXP3 efficiently suppressed Il2 gene expression in HEK 293 cells. HEK 293 cells were transfected with IL2 luciferase reporter vector (kindly provided by Dr J.F. Peyron (Facultè de Medicine Pasteur, Nice) and cotransfected with HA-tagged RelA or FOXP3 or FOXP3ΔE251 expression plasmids where indicated. Results given are the mean ± SD of luciferase light units normalized for Renilla luciferase of the same sample. Results are representative of five independent experiments performed in triplicate. (TIF) [file pone.0048303.s002.tif]

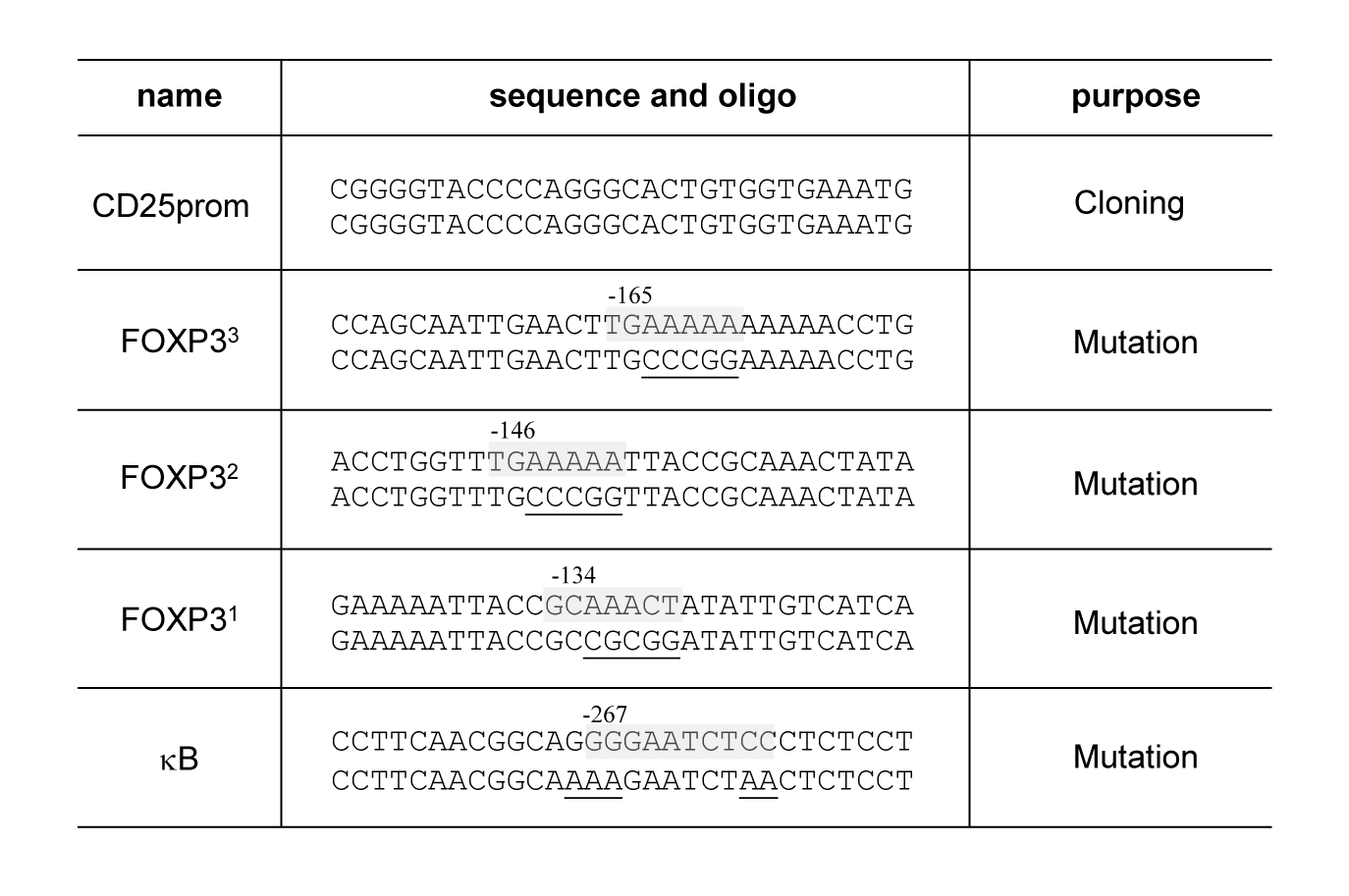

Supplement: Table S1 — Wild type FOXP3 binding sites sequences and mutated primers employed. Primers CD25 introduced a restriction enzyme recognition site for KpnI or XhoI. The primers used for mutational analysis are also shown. In the upper lane, FOXP3 and κB binding sites are evidenced in grey and indicated by position. The primers used for mutational analysis are shown in the lower lane and the underlined letters denote mutated nucleotides. FOXP32/3 mutant was obtained by site-directed mutagenesis using FOXP32 and FOXP33 primers. (TIF) [file pone.0048303.s003.tif]
